# Transcriptional Read-Through Induction Treatment Trial in Intestinal Failure Induced by an *EpCAM* Nonsense Mutation

**DOI:** 10.1155/2012/173195

**Published:** 2012-09-09

**Authors:** Mamata Sivagnanam, James L. Mueller, Reka Szigeti, G. S. Gopalakrishna, Richard Kellermayer

**Affiliations:** ^1^Division of Gastroenterology, Hepatology and Nutrition, Department of Pediatrics, University of California San Diego, La Jolla, Rady Children's Hospital, San Diego, CA 92123, USA; ^2^Department of Pediatrics, University of California San Diego, La Jolla, San Diego, CA 92123, USA; ^3^Department of Pathology, Baylor College of Medicine, Houston, TX 77030, USA; ^4^Section of Pediatric Gastroenterology, Hepatology and Nutrition, Department of Pediatrics, Baylor College of Medicine, Houston, TX 77030, USA; ^5^Texas Children's Hospital, Houston, TX 77030, USA

## Abstract

Congenital tufting enteropathy (CTE) is a rare autosomal recessive diarrheal disorder where epithelial tufts can be present from the duodenum to the large intestine. CTE has been linked to mutations in the epithelial cell adhesion molecule gene (*EpCAM*) Sivagnanam et al. (2008). We recently reported the first case with a nonsense mutation in *EpCAM* Sivagnanam et al. (2010). Here, we explored the clinical and molecular effects of enterally administered gentamicin in this CTE patient. Altogether, our findings indicate that the therapy employed was insufficient to produce notable read-through induction of the *EpCAM* premature termination codon. This report highlights the utility of genetic testing not only in respect of diagnostics, prognostics, and family planning, but potential mutation-specific therapeutic considerations as well.

## 1. Introduction

 Pathogenic nonsense (premature stop) mutations have been detected in over 1800 genetic diseases. The possible therapeutic application of aminoglycosides in such disorders by inducing read-through of the nonsense mutation and promoting the production of full-length functional protein was introduced in 1996 [1]. Aminoglycosides act on specific ribosomal *18s rRNA* residues and can promote misreading of sense codons and read-through of stop codons. Aminoglycoside-induced nonsense-mutation (or premature termination codon/PTC) read-through has been utilized with varying success in dominant and recessive diseases [2]. The potential for this approach as a therapeutic modality is exemplified by an ongoing phase 3 clinical trial with ataluren (an orally bioavailable drug engineered to induce translational read-through) for the treatment of nonsense mutation cystic fibrosis (http://www.clinicaltrials.gov/ct2/show/NCT00803205?term=PTC124+CF&rank=2/). 

However, the pharmacogenetic prospective of aminoglycosides in other nonsense-mediated congenital disorders primarily affecting the gastrointestinal tract has not been explored.

Congenital tufting enteropathy (CTE) is a rare autosomal recessive diarrheal disorder where epithelial tufts can be present from the duodenum to the large intestine. The disease carries significant morbidity and the current therapy for CTE is limited to lifelong parenteral nutrition or small bowel transplantation. CTE has been linked to mutations in the epithelial cell adhesion molecule gene (*EpCAM*) [3]. We recently reported the first case with a nonsense mutation in *EpCAM* [4]. Here, we explored the clinical and molecular effects of enterally administered gentamicin in this CTE patient.

**Figure 1:**
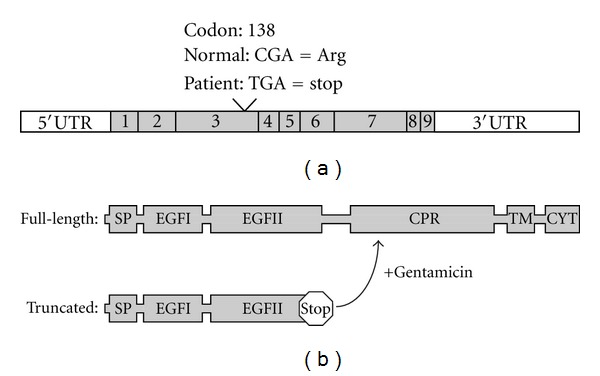
*Schematic depiction of the EpCAM mutation and its consequences.* (a) *EpCAM mRNA* (top): nonsense mutation seen in this patient located in exon 3, codon 138. Shaded boxes represent nine exons with surrounding untranslated (UTR) regions. (b) Full-length EpCAM protein (top) depicting domains: signal peptide (SP), EGF-like (EGF-I and EGF-II), cysteine poor region (CPR), transmembrane domain (TM), and cytoplasmic (CYT) domains, and truncated EpCAM (below) resulting from nonsense mutation and shift towards full length with gentamicin treatment.

The patient presented at 1 month with profuse watery diarrhea, emesis, and failure to thrive. Initial duodenal biopsies obtained by esophagogastroduodenoscopy revealed epithelial tufting consistent with congenital tufting enteropathy. As reported previously [4] genetic testing showed a homozygous nonsense mutation (c.412C > T/p.R138*) in exon 3 of *EpCAM* (Figure 1). The patient has had multiple central line infections requiring hospital admissions. Her stools at baseline were loose and occurred 2–5 times daily. She had 2–6 episodes of emesis a day. At 30 months of age the drug trial was initiated with maintenance of baseline total parenteral nutrition and low rate gastric tube (GT) feedings (10 ml/hr).

## 2. Materials and Methods

The Institutional Review Board of Baylor College of Medicine approved the clinical research protocol. Following informed consent the family recorded daily stool and emesis numbers for 3 weeks when a baseline upper endoscopy was performed and duodenal biopsies were obtained. After the procedure, gentamicin (40 mg/mL) 50 mg/kg/day (the highest dose applied enterally for infectious diarrhea [5]) divided 4 times daily was given to the patient for 3 weeks by GT. At the end of the 3-week gentamicin course a repeat endoscopy with duodenal biopsy was performed. Thereafter, gentamicin was stopped, but clinical recordings were continued. Finally, as an antibiotic control, metronidazole 100 mg twice daily was also given to the patient 10 days after the completion of gentamicin. 

Hematoxylin-eosin (H&E), and immunofluorescent staining of formaldehyde-fixed, paraffin-embedded duodenal biopsy tissue was performed using techniques previously described [3].

For Western blot analysis one piece of flash frozen duodenal tissue collected from the patient before treatment, one after treatment and one normal control were collected and processed as previously described [4].

## 3. Results and Discussion

**Figure 2:**
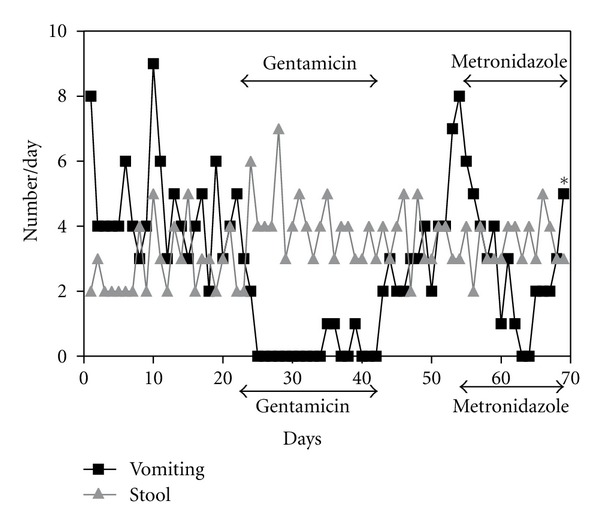
*The clinical response of the patient to enteral gentamicin therapy.* Duodenal biopsies were obtained on day 21, before the initiation of gentamicin, and on day 42, the last day of the therapy. Metronidazole was given 12 days after gentamicin to assess for the effect of an antibiotic frequently used to treat small bowel bacterial overgrowth, but without pharmacogenetic potential.

The patient responded with resolution of her emesis 6-7 days following the initiation of gentamicin. Her stools remained loose and were similar in number to baseline (Figure 2). Her vomiting rapidly recurred after the cessation of gentamicin. The emesis gradually worsened and metronidazole, a control antibiotic therapy, was initiated. Thereafter, the emesis decreased, but for a much shorter period than with gentamicin. On the tenth day of metronidazole therapy vomiting worsened and abdominal distension developed. Five days later, she visited the hospital emergency room and was admitted for dehydration. Therefore, gentamicin had a more sustained beneficial effect for decreasing upper gastrointestinal symptoms (vomiting) in the patient than metronidazole. However, H&E staining showed the same degree of villous blunting with similar level of inflammation both before and after gentamicin. Immunofluorescent staining did not reveal an increase of epithelial cell EpCAM after the therapy (Supplementary Figure 1 of Supplementary Material available online at doi:10.1155/2012/173195). Western blot analysis did not detect any notable change in the full length, unglycosylated form of EpCAM (not shown). 

Since our first report, another CTE patient has been characterized with a nonsense mutation in *EpCAM* [6]. Interestingly, this premature stop codon is located in exon 3, similar to the defect seen in our patient. The gentamicin trial of this report showed promising effects in regards to the clinical response. However this outcome was nonspecific and could be attributed to other aspects (antimicrobial for instance) of the therapy rather than translational read-through induction. Histologic, immunohistochemical, and Western blotting investigations did not reveal any beneficial effects of gentamicin towards alleviating tissue damage or the production of full-length EpCAM. Altogether, our findings indicate that the therapy employed was insufficient to produce notable read-through induction of the *EpCAM* PTC. This negative finding may be a consequence of significant gentamicin dilution by intestinal contents in the small and large bowel. 1 mg/mL of topical gentamicin has been therapeutically effective in a nonsense mutation-associated skin disorder [7]. However, the original 40 mg/mL dose of gentamicin utilized in this study was likely diluted by several magnitudes secondary to the volume and surface characteristics of the human intestinal tract. Unfortunately, the expression of *EpCAM* in the gastric mucosa was not evaluated, but the resolution of vomiting secondary to the experimental therapy could argue for the beneficial effects of gentamicin in the proximal intestine of the patient. It is also possible that mucus and fluid flow from the mucosal crypts into the lumen prevented gentamicin from reaching the intestinal stem cells. Future studies using intravenous gentamicin may be useful, although renal and ototoxicity must be considered. Lastly, we cannot rule out that gentamicin per se is ineffective in producing read-through of this particular PTC in *EpCAM*. 

## 4. Conclusion

Despite the negative results, this report highlights the utility of genetic testing not only in respect of diagnostics, prognostics, and family planning, but potential mutation-specific therapeutic considerations as well. While gene and stem-cell-based treatment strategies are eagerly awaited, translational read-through induction may be a feasible modality for premature-stop-mutation-mediated congenital diarrheal disorders with significant morbidity. However, this therapeutic approach requires careful consideration and testing in a case-by-case fashion prior to implementation.
